# A Novel Pain Relief Approach for the Treatment of Multiple Dental Caries and Pulpitis

**DOI:** 10.7759/cureus.21723

**Published:** 2022-01-30

**Authors:** Yuki Kojima, Ryozo Sendo

**Affiliations:** 1 Anesthesiology, Asahi General Hospital, Asahi, JPN; 2 Anesthesiology, Imakiire General Hospital, Kagoshima, JPN

**Keywords:** ultrasound-guided, non-steroidal anti-inflammatory, inferior alveolar nerve block, dental caries, anesthetics

## Abstract

Anxiety and stress toward treatment can hamper treatment completion in patients with dental caries and pulpitis. Therefore, effective management of post-treatment pain is important because poor pain management can lead to patient dissatisfaction. Ultrasound-guided nerve blocks provide good postoperative analgesia in maxillofacial surgeries. These surgeries can be performed under general or local anesthesia without complications. Here, we present the case of a patient with dental phobia who was successfully treated with these techniques. The patient was a 22-year-old woman with a history of manic-depressive illness who presented with 23 decayed teeth. She had previously undergone vital pulp therapy; however, post-treatment pain led to treatment-related stress, and the patient discontinued the dental treatment. She preferred the dental treatment to be completed with as little pain as possible and wanted to avoid a pulpectomy. The patient’s history of heavy use of non-steroidal anti-inflammatory drugs (NSAIDs) made her resistant to NSAIDs. As a result, the analgesic effect of NSAIDs could not be expected. After intravenous midazolam and propofol sedation, an ultrasound-guided inferior alveolar nerve block was performed bilaterally, and 0.375% ropivacaine was used as a local anesthetic. The patient did not complain of post-operative pain, and no post-operative analgesics were required.

## Introduction

Dental caries and pulpitis are the most common dental diseases requiring treatment. Patients may develop pain and anxiety during the treatment period, potentially reducing their motivation for treatment completion. Therefore, patients afraid of dental treatment often visit the dental clinic after caries when the periodontal disease had already progressed to a severe state. Patients with a pre-existing mental illness may have a strong tendency to develop anxiety associated with dental treatment. Stress management in patients with multiple carious lesions who are unable to continue dental treatment often becomes mandatory for successful outcomes. In particular, effective management of post-treatment pain is an important concern because poor pain management can lead to patient dissatisfaction.

Recently, Kumita et al. reported on the techniques of ultrasound-guided trigeminal nerve block (TNB), ultrasound-guided inferior alveolar nerve block (IANB), and ultrasound-guided maxillary nerve block (MNB) [1]. Some reports have shown that ultrasound-guided TNB provides good post-operative analgesia in maxillofacial surgeries [2-9]. Additionally, ultrasound-guided TNB can be performed under general or local anesthesia without complication [10]. Here, we report the results of our use of this technique via the case of a patient with dental phobia.

## Technical report

The following case report was approved by the patient who provided written informed consent before publication. A 22-year-old woman with a history of manic-depressive illness presented with sharp pain in multiple teeth. An intraoral examination revealed that the patient had severe carious lesions affecting #34, #35, #36, #37, and #47. Owing to poor oral hygiene practices, the patient had developed pericoronitis in #48. Her routine medications comprised olanzapine, trazodone, sodium valproate, brotizolam, eszopiclone, diazepam, belsomra, and lorazepam for the treatment of manic-depressive illness. Additionally, the patient had been taking loxoprofen, diclofenac sodium, and other non-steroidal anti-inflammatory drugs (NSAIDs) for toothache for more than six months, resulting in loss of analgesic effect. In the past, vital pulp therapy was initiated for #46; however, the patient discontinued the treatment because she developed dental phobia due to pain during treatment. In response to her dental phobia, the patient requested that any follow-up dental treatment be completed as non-invasively as possible and without further pulpectomy.

**Figure 1:**
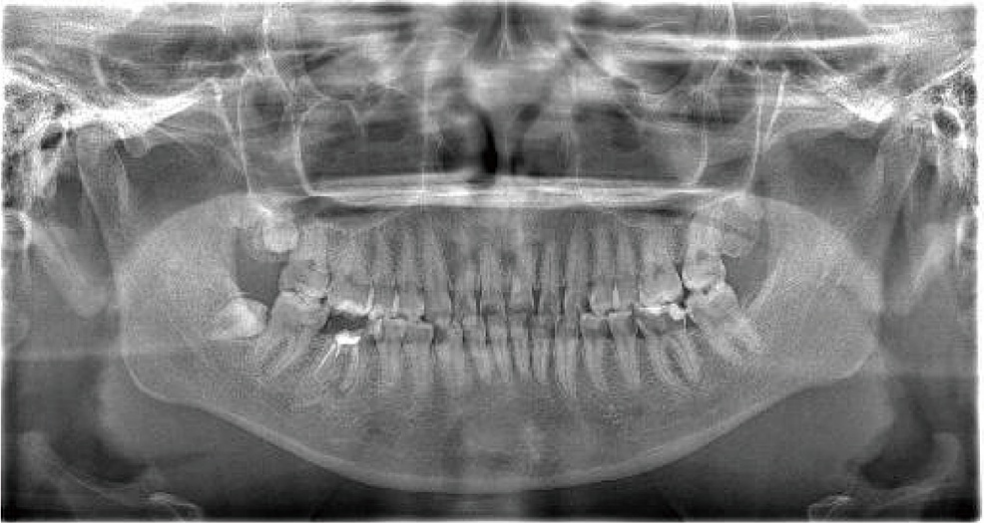
Panoramic radiograph.

**Figure 2:**
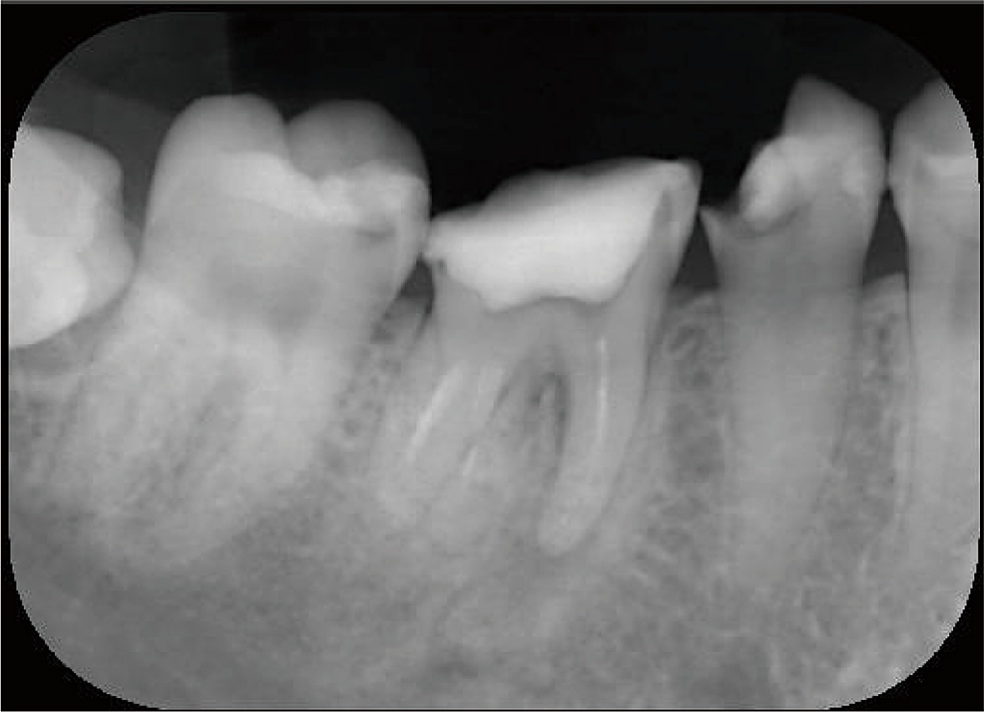
Dental radiograph of #37.

**Figure 3:**
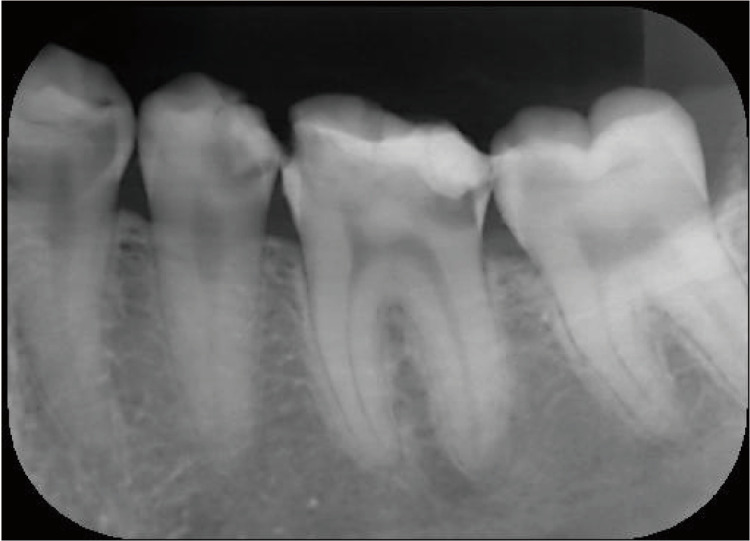
Dental radiograph of #45, #46, and #47.

Intraoral findings by visual inspection showed caries accompanied by a large loss of dentin and enamel, with these findings classified as C2-C3. A panoramic radiograph was obtained to visualize the full jaw (Figure 1). In particular, imaging revealed deep caries at #34, #35, #36, and #37, reaching the pulp (Figures 2, 3), as well as a total of 23 decayed teeth in the patient’s mouth.

The clinical staff initially decided to prioritize the treatment of severe decayed teeth and pericoronitis. The initial treatment was wisdom tooth extraction and caries treatment of both lower jaw molars (#34, #35, #36, #37, #47, and #48). After intravenous sedation with midazolam and propofol for dental phobia, ultrasound-guided IANB was performed bilaterally. A local anesthetic dose of 0.375% ropivacaine 6 mL was administered bilaterally, followed by intravenous administration of 1 g cefazolin sodium before surgical tooth extraction.

**Figure 4:**
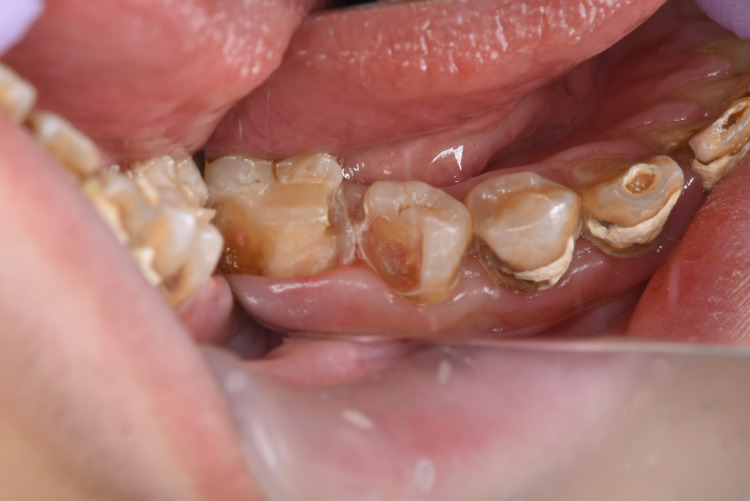
Oral photograph after excavation of caries. Pulpal exposure was identified at multiple points.

The dental pulp was exposed at multiple points (#35, #36; Figure 4) when the caries were completely removed. These areas of pulp exposure were lined by calcium hydroxide, and a glass ionomer restoration was performed for each tooth.

The level of pain relief was assessed after the patient awakened from intravenous sedation and again after going home. There were no complaints of post-treatment pain, and no analgesics were required after the treatment. Pulpitis had not recurred at the eight-month follow-up, and the patient’s recovery was good.

## Discussion

Dentists must be able to diagnose the source of pain and devise strategies for its management [11]. Post-surgical dental pain is commonly moderate to severe. Opioid and non-opioid monotherapy is sometimes used [12]; however, a combination of both (opioid plus acetaminophen or an NSAID) is rarely used in dental practice.

In this case, we treated a patient with dental phobia and multiple severely decayed teeth. It is a logical assumption that patients would hope to undergo dental treatment with minimal pain and, ideally, without needing to take post-operative NSAIDs. In this case, we sought to avoid opioid analgesia because the patient tended to take analgesics frequently. We also felt that it was important to proceed with treatment in a manner that hewed as closely as possible to the patient’s wishes.

With the use of IANB, good post-operative analgesia was obtained after tooth extraction and caries treatment. Post-operative pain management after 48-72 hours is achieved with the analgesic effect, thus leading to less post-treatment pain [7]. When the analgesic options are limited, local anesthesia can be applied due to its ease of use and history of fewer complications. Kumita et al. suggested that the anesthetic agent solution reaches the inferior alveolar nerve, lingual nerve, and buccal nerve when 5 mL of local anesthetic is administered [13]. Therefore, it is likely that the amount used in our patient was sufficient to generate an analgesic response in the pulp of all mandibular teeth.

Two ideas about inflammation have been proposed for the progression of pulpitis. First, inflammation causes an increase in pulp pressure and leads to necrosis of the entire pulp [14-21]. Second, inflammatory substances that are generated from the pulp become necrotic with bacterial invasion [22]. Pulp necrosis is ensured either due to bacterial invasion or the presence of inflammatory mediators. Because pain is a factor that exacerbates inflammation, good analgesia with IANB may have prevented the exacerbation of pulpitis. Furthermore, bacterial invasion can be prevented by the administration of antibiotics and the removal of mechanical caries. We hypothesized that the dental pulp could be preserved without pulpectomy because it was able to incidentally stop the progression of pulpitis. Additionally, it was suggested that administration of antibiotics and complete removal of caries would entirely remove the invading bacteria and that pain management by IANB in pulpitis could preserve the pulp without pulpectomy.

## Conclusions

We consider our success in this area with our patient to be at least somewhat because even though the pulp was infected with bacteria, the infected part was localized. The patient’s young age also proved beneficial in that her youth was directly related to her high pulp activity. We suggest that IANB can be effective in managing post-treatment pain in dental treatments. However, its application in dental treatment is not yet common; therefore, further studies are needed.

## References

[1] Kumita S, Murouchi T, Arakawa J (2017). Ultrasound-guided maxillary and inferior alveolar nerve blocks for postoperative analgesia in gnathoplasty. Asian J Anesthesiol.

[2] Nader A, Schittek H, Kendall MC (2013). Lateral pterygoid muscle and maxillary artery are key anatomical landmarks for ultrasound-guided trigeminal nerve block. Anesthesiology.

[3] Bouzinac A, Tournier JJ, Dao M, Delbos A (2014). Ultrasound-guided maxillary nerve block in adults: feasibility and efficiency for postoperative analgesia after maxillary osteotomy. Minerva Anestesiol.

[4] Allam AE, Khalil AA, Eltawab BA, Wu WT, Chang KV (2018). Ultrasound-guided intervention for treatment of trigeminal neuralgia: an updated review of anatomy and techniques. Pain Res Manag.

[5] Nader A, Kendall MC, De Oliveria GS, Chen JQ, Vanderby B, Rosenow JM, Bendok BR (2013). Ultrasound-guided trigeminal nerve block via the pterygopalatine fossa: an effective treatment for trigeminal neuralgia and atypical facial pain. Pain Physician.

[6] Kumar A, Sinha C, Kumar A, Kumari P, Mukul SK (2018). Ultrasound-guided trigeminal nerve block and its comparison with conventional analgesics in patients undergoing faciomaxillary surgery: randomised control trial. Indian J Anaesth.

[7] Kojima Y, Murouchi T, Akiba M, Oka T (2020). Ultrasound-guided inferior alveolar nerve block for postoperative analgesia after mandibular sequestrectomy: a single-center retrospective study. J Clin Anesth.

[8] Chiono J, Raux O, Bringuier S, Sola C, Bigorre M, Capdevila X, Dadure C (2014). Bilateral suprazygomatic maxillary nerve block for cleft palate repair in children: a prospective, randomized, double-blind study versus placebo. Anesthesiology.

[9] Kojima Y, Furuse K, Murouchi T, Hirabayashi K, Kato M, Oka T (2020). Ultrasound-guided local anesthetic nerve blocks in a forehead flap reconstructive maxillofacial procedure. Anesth Prog.

[10] Kojima Y, Sendo R, Ohno S, Sugimura M (2020). Ultrasound-guided inferior alveolar nerve block for trismus during dental treatment: a case report. JA Clin Rep.

[11] Hargreaves K, Abbott PV (2005). Drugs for pain management in dentistry. Aust Dent J.

[12] Pergolizzi JV, Magnusson P, LeQuang JA, Gharibo C, Varrassi G (2020). The pharmacological management of dental pain. Expert Opin Pharmacother.

[13] Kumita S, Sawada A, Tokura TA (2021). Injectate spread in ultrasound-guided inferior alveolar nerve block: a cadaveric study [In Press]. J Anesth.

[14] Johnson RH, Dachi SF, Haley JV (1970). Pulpal hyperemia--a correlation of clinical and histologic data from 706 teeth. J Am Dent Assoc.

[15] Garfunkel A, Sela J, Ulmansky M (1973). Dental pulp pathosis. Clinicopathologic correlations based on 109 cases. Oral Surg Oral Med Oral Pathol.

[16] Tyldesley WR, Mumford JM (1970). Dental pain and the histological condition of the pulp. Dent Pract Dent Rec.

[17] Guthrie TJ, Mcdonald RE, Mitchell DF (1965). Dental pulp hemogram. J Dent Res.

[18] Hasler JE, Mitchell DF (1970). Painless pulpitis. J Am Dent Assoc.

[19] Koch G, Nyborg H (1970). Correlation between clinical and histological indications for pulpotomy of deciduous teeth. J Int Assoc Dent Child.

[20] Dummer PM, Hicks R, Huws D (1980). Clinical signs and symptoms in pulp disease. Int Endod J.

[21] Seltzer S, Bender IB, Ziontz M (1963). The dynamics of pulp inflammation: correlations between diagnostic data and actual histologic findings in the pulp. Oral Surg Oral Med Oral Pathol.

[22] Ricucci D, Bergenholtz G (2004). Histologic features of apical periodontitis in human biopsies. Endod Topics.

